# MASP-1 and MASP-2 Serum Levels Are Associated With Worse Prognostic in Cervical Cancer Progression

**DOI:** 10.3389/fimmu.2018.02742

**Published:** 2018-11-23

**Authors:** Carlos Afonso Maestri, Renato Nisihara, Hellen Weinschutz Mendes, Jens Jensenius, Stephen Thiel, Iara Messias-Reason, Newton Sérgio de Carvalho

**Affiliations:** ^1^Liga Paranaense de Combate ao Câncer, Erasto Gaertner Hospital, Curitiba, Brazil; ^2^Department of Medicine, Positivo University, Curitiba, Brazil; ^3^Immunopathology Laboratory, Department of Clinical Pathology, Federal University of Parana, Curitiba, Brazil; ^4^Department of Biomedicine, Aarhus University, Aarhus, Denmark; ^5^Department of Gynecology, Clinical Hospital, Federal University of Parana, Curitiba, Brazil

**Keywords:** complement, lectin pathway, HPV, cervical intraepithelial neoplasia, cervical cancer

## Abstract

**Background:** MBL-associated serine proteases (MASP-1, MASP-2, MASP-3, MAp-44, and MAp-19) are key factors in the activation of the lectin pathway of complement. Serum levels of these components have been associated with recurrence and poor survival of some types of cancer, such as colorectal and ovarian cancer. In this investigation, we determined the serum levels of MASP-1, MASP-2, MASP-3, MAp-44, and MAp-19 in patients with cervical cancer and cervical intraepithelial neoplasia (CIN).

**Methods:**A total of 351 women who underwent screening for cervical cancer or treatment at the Erasto Gaertner Cancer Hospital in Curitiba-Brazil, were enrolled in the study. Based on their latest cervical colposcopy-guided biopsy results, they were divided into four groups: CIN-I: *n* = 52; CIN-II: *n* = 73; CIN-III: *n* = 141; and invasive cancer: *n* = 78. All the serum protein levels were determined by time-resolved immunofluorometric assay (TRIFMA).

**Results:**Patients with invasive cancer presented significantly higher MASP-2, MASP-1, and MAp-19 serum levels than other groups (*p* < 0.0001; *p* = 0.012; *p* = 0.025 respectively). No statistically significant differences in MASP-3 and MAp-44 serum levels were found between the four studied groups. In addition, high MASP-2, MASP-1, and MAp-19 serum levels were significantly associated with poor survival in patients with invasive cancer and relapse (*p* = 0.002, *p* = 0.0035 and *p* = 0.025, respectively).

**Conclusion:**High MASP-2, MASP-1, and MAp-19 serum levels were associated with cervical cancer progression and worse disease prognosis. These novel findings demonstrate the involvement of the serine proteases of the lectin pathway in the pathogenesis of cervical cancer and future investigations should clarify their role in the disease process.

## Introduction

Human papilloma virus (HPV) is amongst the most common worldwide viral infection transmitted sexually. And although most HPV infections generally clear up on its own, a small percentage of these infections caused by specific HPV types can persist and evolve to cancer. The highest prevalence rate of HPV is observed in women younger than 25 years and decreases at later ages, but in many populations, there is a secondary peak in peri-menopause (1, 2). Young infected patients can usually eliminate the virus without presenting clear clinical evidence, due to an effective cell-mediated immune response causing the lesions to regress. The inability to develop an effective immune response to clear or control infection results in persistent infection that, in the case of oncogenic HPVs, creates a greater likelihood of progression to high-grade cervical intraepithelial neoplasia (CIN-2, CIN-3) and carcinoma. On the other hand, about 50% of HPV infections in women with normal cytology will resolve in less than 1 year, and approximately 90% of women with either normal cytology or CIN-1 diagnoses will ultimately resolve on their own (2).

It is known that HPV has a variety of strategies to evade the immune response, and when this occurs, HPV replication continues leading to persistent infection (1, 2). Among the approaches used by HPV to evade the host immune response are suppression of important inflammatory/immunological pathways that enable virus escaping from host immune surveillance (3). Due to suppression of some danger signals, HPV infection does not produce cytolysis, cytopathic cell death or interferon (IFN) release, leading to lower grade inflammation (1, 4, 5).

Chronic inflammation in the tumor microenvironment and evasion of the antitumor effector immune response are two of the hallmarks required for oncogenesis and cancer progression. The innate immune system not only plays a critical role in perpetuating these tumor-promoting hallmarks but also in developing antitumor adaptive immune responses (6). Thus, understanding the dual role of the innate system in cancer immunology is required for the design of combined immunotherapy strategies able to tackle established tumors (6). The lectin pathway of the complement system is part of innate response, being triggered when pattern recognition molecules (PRMs), including the two collectins [mannan-binding lectin (MBL) and collectin-LK] and three ficolins (ficolin-1,-2, and-3) initiate the complement activation upon binding to carbohydrates present on the surfaces of microbes or altered tissues. When this occurs, three MBL-associated proteases (MASP-1,-2, and-3) provide activation of the complement system, and two MBL-associated proteins (MAp44 and MAp19) serve as natural endogenous competitive inhibitors (7). The proteins MASP-1, MASP-3, and Map44 arise from the *MASP1* gene by mutually exclusive splicing (8). In a similar mode, MASP-2 and MAp19 arise from the *MASP2* gene (7). All these components are key factors in the activation of the lectin pathway of complement. Serum levels of these components were associated with recurrence and poor survival of some types of cancer such as colorectal or ovarian cancer (9, 10). Increased serum levels of MBL and MASP-2 were found in patients with colorectal cancer, which were not explained by genetic profiles (9, 10). So far, no study has addressed the serine proteases of the lectin pathway proteins in relation to cervical intraepithelial neoplasia and carcinoma. Our research group had previously reported on the MBL concentrations in women presenting with HPV-associated cervical lesions, showing there was no statistically significant difference between the median serum MBL concentrations in women presenting with CIN-1, CIN-2, CIN-3 lesions or invasive cervical cancer (11).

The aim of the present study was to investigate whether MASP-1, MASP-2, MASP-3, MAp-44, and MAp-19 serum levels are involved in the pathogenesis and progression of cervical intraepithelial neoplasia by measuring their concentrations in women presenting moderate grade of cervical intraepithelial neoplasia and cervical invasive cancer.

## Subjects and methods

This cross-sectional study was carried out at the Erasto Gaertner Cancer Hospital (HEG) in Curitiba, southern Brazil, which is a reference center for the treatment of gynecological malignancies. The Institutional Ethics Committee approved the study.

All subjects were followed at the out-patient clinic of the HEG and were consecutively included from January 2011 to March 2012. Inclusion criteria were cervical cancer screening and treatment at HEG. We also collected historical data on disease progression from patients' medical records. Exclusion criteria were pregnancy, HIV-positivity, systemic infection, autoimmune disease, and blood transfusion within the last 60 days. Written informed consent was obtained from all patients.

A group of 344 women was included in this study. Based on their latest cervical colposcopy-guided biopsy, the subjects were divided into four groups: low grade CIN-1: *n* = 52 (control group); moderate CIN-2: *n* = 73; CIN-3: *n* = 141; and invasive cancer (Ca): *n* = 78. The loop electrical excision procedure or cold-knife cone excision was used to confirm the previous biopsy results. A 3-ml sample of venous blood was collected from each subject and allowed to coagulate. The coagulated blood was centrifuged, and the serum was separated, aliquoted (500 μl), and stored at −80°C, until use for MASP-1, MASP-2, MASP-3, Map-44, and Map-19 concentration determinations.

The concentrations of MASP-1, MASP-2, MASP-3, Map-44, and Map-19 were measured in according to published previously (8, 12, 13). The assays used for protein determinations were monoclonal antibody-based time-resolved immunofluorometric assays (TRIFMAs) and were carried out in partnership with the Institute of Medical Microbiology and Immunology, University of Aarhus, Aarhus. The human MASP-1 assay was based on competition from MASP-1 in serum with the interaction between anti-MASP-1 antibody and a fragment of MASP-1 coated onto microtitre wells. In the case of MASP-2 concentration (“sandwich ELISA”), rat anti-MASP-2 mAb (clone 8B5) was used for coating while biotinylated rat anti-MASP-2/MAp19 mAb (clone 6G12). MASP-3, Map-44, and Map-19 was determined by sandwich ELISA using specific antibodies. All the assays used Eu^3+^-labeled streptavidin (Perkin Elmer, USA)—for detection. All the antibodies were kindly provided by Prof. Jens C. Jensenius (Aarhus University, Denmark).

Data were analyzed as frequency and contingency tables. The Kolmogorov–Smirnov test was used to assess the data distribution. Central tendency was expressed as mean and standard deviation or median and interquartile range (IQR), and the Mann–Whitney test was used to compare numerical data. A receiver-operator characteristic curve (ROC) was also calculated for MASP-2. Results were considered statistically significant at *p* < 0.05. GraphPad Prism 7.0 software was used for statistical analysis.

## Results

The serum concentration of MASP-1, MASP-2, MASP-3, MAp-44, and MAp-19 proteins in the investigated groups is presented in Table 1. Significantly higher MASP-2, MASP-1, and MAp-19 serum were observed in patients with invasive cancer compared to other groups (*p* < 0.025). However, no significant differences were observed for MASP-3 and MAp-44 levels.

**Table 1:** MASP-1, MASP-2, MASP-3, MAp-44, and MAp-19 serum concentrations in patients with cervical lesions and cancer.

	**CIN-1 (*n* = 52)**	**CIN-2 (*n* = 73)**	**CIN-3 (*n* = 141)**	**Ca (*n* = 78)**	***p***
MASP-1 (ng/mL) median (IQR)	5,636 (3,758–8,129)	6,083 (3,823–8,422)	4,714 (3,319–6,839)	6,435 (3,971–9,074)	0.012
MASP-2 (ng/mL) median (IQR)	232.2 (163–386)	264.5 (190–418)	282.5 (204–410)	354.4 (234–570)	0.001
MASP-3 (ng/mL) median (IQR)	3,846 (2,750–5,212)	3,984 (3,181–5,180)	4,053 (2,947–5,071)	3,954 (2,705–5,142)	0.829
Map-44 (ng/mL) median (IQR)	1,695 (1,343–2,248)	1,787 (1,471–2,271)	1,710 (1,434–2,099)	1,866 (1,598–2,171)	0.246
Map-19 (ng/mL) median (IQR)	214.2 (152–289)	239.3 (162–318)	234.4 (167–298)	309.9 (219–487)	0.025

*IQR, Interquartile range; CIN, Cervical intraepithelial neoplasia; Ca, Cancer*.

Regarding clinical evolution, 41 (11.9%; mean age 51.4 years) patients died within 24–48 months. Surviving patients had a mean age of 34.8 years. When serum concentrations were evaluated, median MASP-2 levels were higher among patients who died than among those who survived (439.4 ng/ml × 278.3 ng/mL; *p* < 0.0001). For MASP-1 (6988 ng/mL vs. 5110 ng/mL; *p*=0.0035) and MAp-19 (366 ng/mL vs. 234 ng/mL; *p* = 0.012), the same trend was observed. Serum concentration of MASP-3 and MAp-44 were not significantly different between the two groups. When patients who died and those who experienced relapse of CIN or cancer during follow-up were considered, only MASP-2 and MAp-19 were significantly different (Table 2).

**Table 2:** Association between MASP-1, MASP-2, MASP-3, MAp-44, and MAp-19 serum concentrations and clinical evolution in patients with cervical lesions.

	**Without death or relapse**	**With death or relapse**	P^*^
MASP-1 (ng/mL) median	5,144	6,206	0.069
MASP-2 (ng/mL) median	273.7	382.4	0.002
MASP-3 (ng/mL) median	3,960	3,846	0.58
Map-44 (ng/mL) median	1,739	1,814	0.76
Map-19 (ng/mL) median	239.7	299.5	0.025

* *Mann–Whitney test*.

ROC curve was used to show the sensitivity and specificity for MASP-2 serum concentrations in relation to the prognosis of cervical cancer progression. High levels of MASP-2 (>291.9 ng/mL) showed a sensitivity of 71.3 and a specificity of 63.2 for worse prognosis of cervical lesions.

## Discussion

Our study demonstrates that MASP-2, MASP-1, and MAp-19 could have a role in the progression of cervical cancer. Patients diagnosed with invasive cancer showed a significant increase in the concentrations of these lectin pathway components. In another study conducted by our group, we failed to establish the relationship between serum concentration of MBL and the evolution of cervical intraepithelial neoplasias (10). These novel findings indicate possible involvement of the lectin pathway in the immunopathogenesis of cervical cancer, although it can result, in part, from activation of the complement system due to the presence of tumor cells. In fact, the participatory role of lectin pathway components in defense against pathogens such as viruses, bacteria, or fungi is much better known and studied: MASPs deficiencies are associated with increased infection susceptibility, and increased levels are associated with tissue injury (14). However, studies about its role in the development of cancer are scarce.

In a study of colorectal cancer, Ytting et al. (10) observed higher MASP-2 serum concentrations in patients with cancer, although no differences for *MASP2* genotypes were detected between patients with colorectal cancer and healthy controls. Furthermore, serum levels of MASP-2 have been associated with post-operative infections, recurrent cancer, and poor survival in colorectal cancer patients (9). These findings suggest that increased MASP-2 serum concentrations may occur due to factors other than genetics and that MASP-2 may play a role in cancer development and disease outcome. Corroborating these observations, in our study high MASP-2 serum concentrations were related to worse prognosis in cervical cancer and interestingly, they were mainly altered in patients who died and/or had disease recurrence. MASP-2 is produced in hepatocytes, and its promoter is regulated by cytokines such as interleukin (IL)-1b and IL-6 or transcription factor STAT3 (15).It is possible that tumor tissue releases mediators that may increase serum levels of MASP-2, however this is a hypothesis that needs to be clarified. Studying genetic polymorphism and MASP-2 serum levels in patients with ovarian cancer, Swierzko et al. (9) concluded that the expression of MBL and MASP-2 is altered in ovarian cancer, possibly indicating involvement of the lectin pathway of complement in the disease. Similar results of increased MASP-2 serum levels were described in children with tumors of the central nervous system (16) and in patients with acute lymphoblastic leukemia and non-Hodgkin's lymphoma (16).

In our study, MASP-1 serum concentrations were significantly higher in cancer patients than in other studied groups. The normal MASP-1 concentration in serum/plasma is approximately 20-fold higher than that of MASP-2 (13). It is known that MASP-1 activates MASP-2 in heterocomplexes of large oligomeric MBL and produces 60% of the C2b responsible for C3 convertase formation (17). Additionally, MASP-1 was reported to be essential for the development of autoimmune-associated inflammatory tissue injury by activating the alternative complement pathway in an experimental model of inflammatory arthritis (18). There are no studies on MASP-1 concentrations in cancer.

Complement system and macrophages collaborate synergistically to maintain progression of angiogenesis, and create conditions of carcinogenesis such as dysregulation of mitogenic signaling pathways, cellular proliferation, angiogenesis, resistance to apoptosis, and escape from immuno-surveillance. Macrophages release mediators that modulate inflammation and acquired immunity response. In addition, promote angiogenesis, tissue remodeling and tissue repair (19). The tumor growth is sustained by the infiltration of M2-tumor-associated macrophages, and high levels of C3a and C5a. Macrophages have receptors for both C3a and C5a on their cell surface, and this specific binding affects the functional modulation and angiogenic properties (19). High levels of MASP-2 and MASP-1 may increase activation of the complement system by the lectin pathway, thereby augmenting the release of C5a, a potent anaphylatoxin that activates cellular responses involved in tumor growth and progression (20). During cervical lesion progression, the number of M2 macrophages is significantly increased. The aggregation of M2 is a key event in the pathological process of carcinogenesis (21). M2 macrophages increase vascular endothelial growth factor (VEGF) and metalloprotease-9 secretion to aid in tissue repair. On the other hand, complement system activation by tumors may result in basement membrane disruption, tumor growth, and metastasis (22). *In vivo*, HPV does not bind directly to cells but requires contact with the basement membrane. This contact can be accomplished by microabrasions on the cervical surface, which reveal the basement membrane. The most plausible receptor of the major capsid protein (L1) of HPV appears to be the tissue-specific heparin sulfate proteoglycan, which belongs to the basement membrane (23, 24). It is possible that patients with higher serum levels of MASP-1 and/or MASP-2 present exacerbate activation of complement leading to the disturbance of the basement membrane, and ultimately to its damage or rupture and consequently, tumor invasion.

Interestingly, HPV protein (E2, E6, E7) action on *IL10* gene leads to increased IL-10 levels which in turn increases HPV E6 and E7 expression, leading to a vicious cycle (3, 22). IL-10 is an anti-inflammatory cytokine that modulates cytokine synthesis and exerts effects on resident and circulating immune cells, suppressing the immune system; however, their role in cancer remains controversial (3). On the other hand, activation of complement system is clearly an inflammatory process that acts to protect the host. However, to date, the majority of studies on cancer and the lectin pathway have shown a relation with worse prognosis when serum levels of components of this pathway were increased (9, 16). Future studies should investigate how complement interacts with the basement membrane and tumor tissue along with the influence of cytokine network on this system.

The limitations of this study are due to its cross-sectional study design, which prevented the use of serial dosages of the lectin pathway components over time to evaluate the stability of component concentrations throughout disease progression. Thus, future investigations evaluating the role of the different components of the lectin pathway, including collectins, ficolins and MASPs in the evolution of cervical lesions should be encouraged. In addition, our study did not include a healthy control group, for obvious reason, since it would be very difficult to obtain a group of women without HPV infection, either for ethical reasons or lack of lab tests to prove this condition. Thus, we believe that CIN-1 was the appropriate comparison group for our study, especially considering that such patients show good immune response against HPV and generally (>90%) do not progress to cancer (2).

In conclusion, our study shows that high MASP-2, MASP-1, and MAp-19 serum levels are associated with cervical cancer progression and worse disease prognosis. Future investigations should clarify the role of these complement components in cervical cancer immunopathogenesis.

## Author contributions

CM and NdC: protocol and project development, data collection or management, and manuscript writing and editing; RN: protocol and project development, data analysis, and manuscript writing and editing; HM: protocol and project development, laboratorial assays, and manuscript writing and editing; JJ, ST, and IM-R: protocol and project development, and manuscript writing and editing.

### Conflict of interest statement

The authors declare that the research was conducted in the absence of any commercial or financial relationships that could be construed as a potential conflict of interest.
